# In-vitro and in-vivo comparisons of high versus low concentrations of inhaled epoprostenol to adult intubated patients

**DOI:** 10.1186/s12931-021-01827-4

**Published:** 2021-08-21

**Authors:** Jie Li, Ashley E. Augustynovich, Payal K. Gurnani, James B. Fink

**Affiliations:** 1 Department of Cardiopulmonary Sciences, Division of Respiratory Care, Rush University, 600 S Paulina St, Suite 765, Chicago, IL 60612 USA; 2 Department of Pharmacy, Rush University Medical Center, Chicago, IL USA; 3 Aerogen Pharma Corp, San Mateo, CA USA

**Keywords:** Inhaled epoprostenol, Concentration, Pulmonary hypertension, Hypoxemia

## Abstract

**Background:**

Inhaled epoprostenol (iEPO) has been shown to reduce pulmonary artery pressure and improve oxygenation. iEPO is mainly delivered via a syringe pump with feed tubing connected to a vibrating mesh nebulizer with high or low formulation concentration delivery.

**Methods:**

An in vitro study and a two-period retrospective case–control study were implemented. The in vitro study compared iEPO delivery via invasive ventilation at low concentrations of 7.5, and 15 mcg/mL and high concentration at 30 mcg/mL, to deliver the ordered dose of 30 and 50 ng/kg/min for three clinical scenarios with predicted body weight of 50, 70 and 90 kg. While in the clinical study, adult patients receiving iEPO via invasive ventilation to treat refractory hypoxemia, pulmonary hypertension, or right ventricular failure were included. 80 patients received low concentration iEPO at multiple concentrations (2.5, 7.5, and 15 mcg/mL, depending on the ordered dose) from 2015 to 2017, while 84 patients received high concentration iEPO at 30 mcg/mL from 2018 to 2019.

**Results:**

In the in vitro study, there were no significant differences in aerosol deposition between high vs low concentrations of iEPO at a dose of 50 ng/kg/min. In the clinical study, age, gender, ethnicity, and indications for iEPO were similar between high and low concentration groups. After 30–120 min of iEPO administration, both delivery strategies significantly improved oxygenation in hypoxemic patients and reduced mean pulmonary arterial pressure (mPAP) for patients with pulmonary hypertension. However, no significant differences of the incremental changes were found between two delivery groups. Compared to low concentration, high concentration delivery group had better adherence to the iEPO weaning protocol (96% vs 71%, p < 0.001), fewer iEPO syringes utilized per patient (5 [3, 10] vs 12 [6, 22], p = 0.001), and shorter duration of invasive ventilation (6 [3, 12] vs 9 [5, 18] days, p = 0.028). Intensive care unit length of stay and mortality were similar between two groups.

**Conclusion:**

Compared to low concentration delivery of iEPO, high concentration iEPO via a vibrating mesh nebulizer maintained clinical benefits and increased clinician compliance with an iEPO weaning protocol, required less medication preparation time, and shortened duration of invasive ventilation.

## Introduction

Inhaled epoprostenol (iEPO), a naturally occurring prostaglandin that acts as vascular smooth muscle relaxant, has been utilized clinically for over two decades and has been shown to reduce pulmonary artery pressure [1–5] and improve oxygenation [3–12]. Despite its clinical significance, an optimal delivery strategy remains unknown.

In the recent years, the use of a vibrating mesh nebulizer (VMN) with continuous external feed has been increasingly utilized to deliver iEPO in-line during invasive ventilation [3–7, 10–12], as it is powered by electricity with no gas added to the ventilator circuit, and it has little to no residual volume. When a VMN is utilized, iEPO is prepared in a 60 mL syringe and the dose is adjusted by setting different pump rates, depending on the formulation concentration to deliver the desired dose, thereby introducing the formulation to the mesh “drop by drop” which produces aerosol consistently but intermittently. A pump rate that matches or exceeds the nebulizer output results in continuous aerosol generation. Thus, there are two major strategies to administer iEPO via VMN: (1) using one high concentration and adjusting the pump rate to deliver a full range of doses. The pump is usually set at a low rate (1–8 mL/h) and the aerosol is generated intermittently [7, 12]. (2) preparing different concentrations of medication, then changing the concentration and setting the pump rate (8–20 mL/h) close to the nebulizer output to produce constant and continuous emitted aerosol [10, 13]. The latter strategy (low concentration) requires a change of formulation concentration and a new syringe for each ordered dose change. Both strategies are utilized in clinical practice with no evidence available to compare the efficiency of aerosol delivery and cost of these two strategies.

At our institution, we started using iEPO via VMN with low concentration strategy in 2015, due to the concerns that iEPO had a short half-life. The concern was if iEPO was not consistently delivered to the patient, the interruption of delivery might cause rebound effects [11]. In our previous in vitro study, we found the output for VMN was 20 mL/h [13], thus we prepared three concentrations of iEPO to deliver different doses while maintaining pump rate at 8–20 mL/h. However, the process of preparing medication, changing the drug concentrations to meet patient needs, and adjusting the pump rate was complicated. This resulted in delays in initiation, response to changing orders, and weaning, especially during the night, weekend and holidays when staffing was limited. We intended to change the delivery strategy to high concentration delivery, but we had concerns whether it could provide a similar aerosol deposition at the low concentration delivery. Therefore, an in vitro study to compare the efficiency of high and low concentration delivery strategies was implemented in the end of 2017, the in vitro study results became the basis to switch to high concentration delivery in January 2018. A retrospective study was implemented to compare the two cohorts using different delivery strategies of iEPO from August 2015 to December 2019. Thus, we aimed to report the aerosol delivery efficiency of the two delivery strategies in the in vitro and in vivo comparisons.

## Methods

### In-vitro study

#### Experimental set-up

**Fig. 1:**
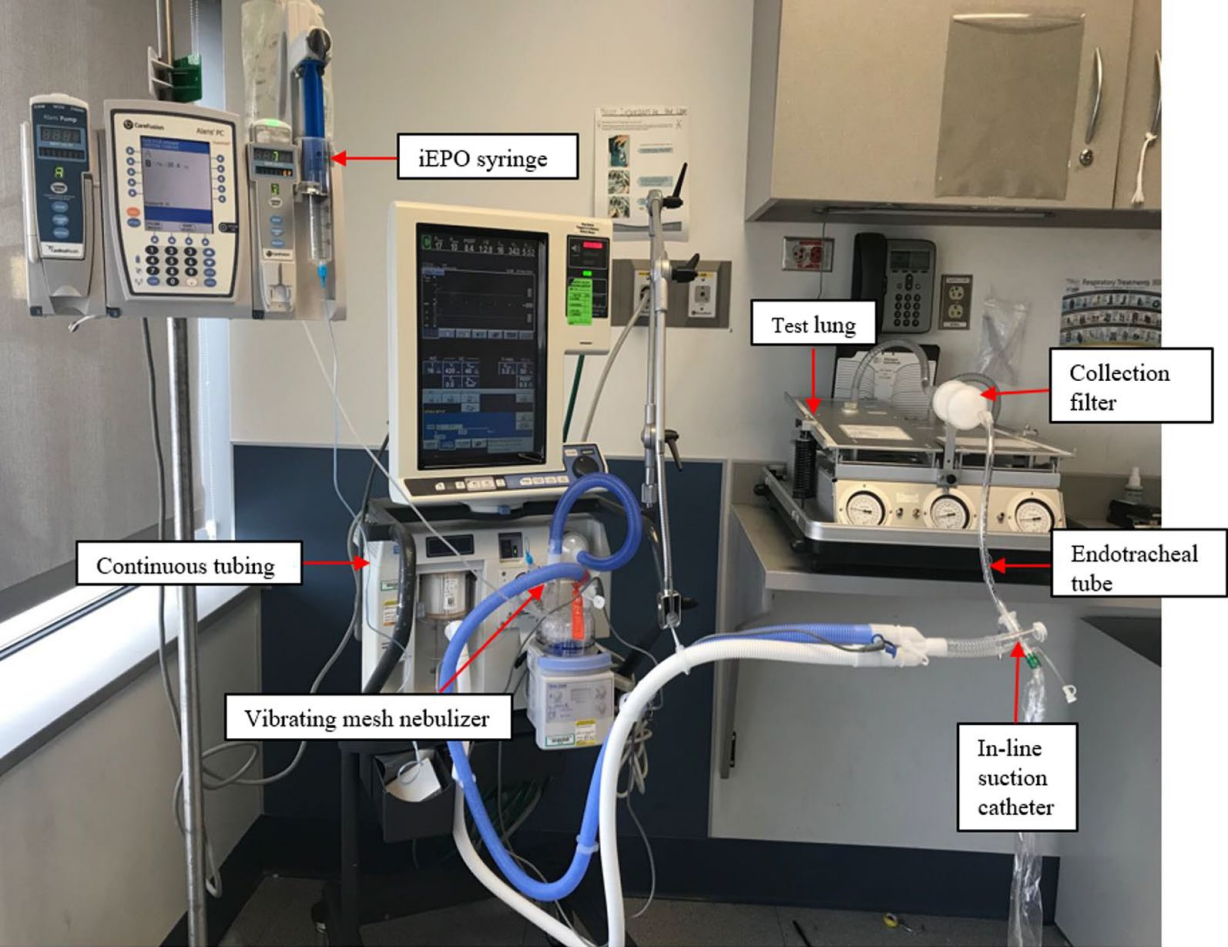
In vitro experiment set up. A collection filter was used to connect the model lung and the endotracheal tube (ID = 8.0 mm) and a dual-limb ventilator. iEPO was prepared in the syringe that was placed at the syringe pump, with the feeding tube connected to a vibrating mesh nebulizer at the inlet of humidifier. *iEPO* inhaled epoprostenol

The in vitro experimental set-up consisted of the critical care ventilator (PB840, Medtronic, Minneapolis, MN) with a humidifier and a heated wire circuit (MR850, Fisher & Paykel, Auckland, New Zealand), an 8.0 mm ID endotracheal tube (Medtronic, Minneapolis, MN) and a closed suction catheter (Kimberly-Clark, Irving, TX) used to connect the Y-piece of the ventilator circuit and a model lung (TTL, Michigan Instruments, Grand Rapids, MI). The collecting filter (AirLife 002446, Carefusion, Yorba Linda, CA) was placed between the distal end of endotracheal tube and the model lung. The VMN (Aerogen Solo, Galway, Ireland) was attached to the inlet of the humidifier. Syringes (CNTS, Aerogen) were filled with 50 mL of the specific concentration of iEPO, attached to the continuous nebulizer tube set (CNTS; Aerogen), manually primed with syringe placed into a pump (Alaris, Carefusion, San Diego, CA) and tubing attached to the VMN (Fig. 1). The passive test lung was set at a resistance of 5 cm H_2_O/L/s and a compliance of 50 mL/cm H_2_O. Ventilator and pump settings were based on three predicted body weights (PBW) of 50, 70, and 90 kg: PRVC, tidal volume (Vt) 6 mL/kg, respiratory rate (RR) 16 breaths/min, positive end-expiratory pressure (PEEP) 5 cm H_2_O, fraction of inspired oxygen (F_I_O_2_) 0.6, and inspiratory time (Ti) 1.0 s.

#### iEPO preparation and doses

iEPO (1.5 g, Veletri, Actelion Pharmaceuticals, San Francisco, CA) was prepared with sterile water to produce concentrations of 7.5, 15, and 30 mcg/mL in 60 mL syringes. Syringe pump rates were set according to the PBW (50, 70, and 90 kg), with doses of 30 and 50 ng/kg/min. In the high concentration group, iEPO at 30 mcg/mL was utilized, regardless of dose and patient’s PBW. In the low concentration group, in order to avoid exceeding the nebulizer output (maximum 20 mL/h), 15 mcg/mL was used to deliver 50 ng/kg/min for the three scenarios while 7.5 and 15 mcg/mL were used to deliver 30 ng/kg/min. Each experimental run lasted for 20 min and repeated three times (n = 3). Between each run, the ventilator circuit was allowed to stabilize for 1 min [13].

#### Assay analysis

At the end of each experimental run, epoprostenol was eluted from the collecting filter with 10 mL of sterile water and were analyzed with UV spectrophotometry at 205 nm [13].

#### Comparisons

We compared aerosol delivery at two target doses: 50 and 30 ng/kg/min. iEPO solution concentrations of 30 mcg/mL and 15 mcg/mL were compared for 50 ng/kg/min, while for 30 ng/kg/min, iEPO concentration of 30 mcg/mL was compared with 15 and 7.5 mcg/mL.

### In-vivo study

#### Study design

This was a retrospective study enrolling adult patients (≥ 18 years) who received iEPO via invasive ventilation at Rush University Medical Center between August 2015 and December 2019. Patients were excluded if meeting any of the following criteria: (1) received a combined treatment of iEPO with inhaled nitric oxide (iNO), intravenous epoprostenol or prone positioning; (2) iEPO for palliative care; (3) iEPO was initiated during extracorporeal membrane oxygenation (ECMO) or cardiac surgical operation; (4) iEPO was used for less than 30 min. This study was approved by our ethic committee (Approval No. 19073005-IRB01). Written consents were not required since this was a retrospective, observational cohort study.

#### iEPO indications

Per our institution protocol, iEPO indications included: (1) Refractory hypoxemia, which was defined as the ratio of partial pressure of arterial oxygen (PaO_2_) to fraction of inspired oxygen (F_I_O_2_) ≤ 200 mm Hg with PEEP at 8 cm H_2_O. (2) Pulmonary hypertension, defined as a mean pulmonary arterial pressure (mPAP) ≥ 30 mm Hg or systolic pulmonary arterial pressure (sPAP) ≥ 40 mm Hg. (3) Right heart failure, defined as central venous pressure (CVP) ≥ 15 mm Hg with cardiac index (CI) < 2.2 L/min/m^2^. Patients may have been classified as having more than one indication.

#### iEPO administration

Per our institution protocol, iEPO was initiated at a dose of 50 ng/kg/min based on PBW and maintained for a minimum of 24 h after initiation. iEPO weaning was determined by the treating physician(s) and titrated down by 10 ng/kg/min every 30–60 min if the patient’s status remained stable [7]. In the high concentration group, iEPO dose was titrated by adjusting the pump rate. In the low concentration group, iEPO dose was titrated by adjusting the pump rate and iEPO concentrations. For example, if the dose was reduced from 40 to 30 ng/kg/min, the iEPO concentration was changed from 15 to 7.5 mcg/mL, in order to maintain pump rate between 8 and 20 mL/h.

#### Data collection

Patient demographics including age, gender, race, diagnosis, and iEPO indication were collected. At 30–120 min before and 30–120 min after iEPO initiation, hemodynamic parameters including mean blood pressure (mBP), mPAP, cardiac output (CO), CI, mixed venous oxygen saturation (SvO_2_), and pulmonary vascular resistance (PVR), heart rate (HR), RR, saturation of pulse oximetry (SpO_2_), PaO_2_, F_I_O_2_, and PEEP were obtained, if available. The use of ECMO, number of iEPO syringes used, iEPO duration, duration of invasive ventilation, length of stay and mortality in the intensive care unit (ICU) were recorded, as well as the compliance with weaning protocol.

#### Outcomes

In patients with refractory hypoxemia, the primary outcome was the change of PaO_2_/F_I_O_2_ ratio or SpO_2_/F_I_O_2_ ratio between the high and low concentration groups. In patients with pulmonary hypertension and/or right heart failure, incremental changes in mPAP were compared between the two groups. Positive response to iEPO was defined as PaO_2_/F_I_O_2_ ratio or SpO_2_/F_I_O_2_ ratio increased by 10% [14] and 20% [4–6], or mPAP decreased by 10% [6]. Secondary outcomes included the iEPO duration, number of iEPO syringes used, adherence with iEPO weaning protocol, duration of invasive ventilation, ICU length of stay and mortality between two groups.

### Statistical analysis

The Kolmogorov–Smirnov test was used to assess the normality of distribution for considered variables. Continuous variables were expressed as mean (standard deviation [SD]) or median (inter-quartile range [IQR]). For the in vitro study, Mann Whitney test was used to compare the inhaled dose of two iEPO concentrations at 30 and 15 mcg/mL, while Kruskal Wallis test was used to compare the inhaled dose of three iEPO concentrations. For the clinical study, variables of pre and post iEPO within the same group were compared with Wilcoxon sign rank test or Paired t-test, whereas one-way analysis of covariance (ANCOVA) was conducted to determine the difference between the high and low concentration delivery for incremental changes in iEPO response, controlling for baseline variables. Differences in categorical variables were assessed with the Chi-square or Fisher exact test. A p-value of < 0.05 was considered to be statistically significant for all tests. Data analysis was conducted with SPSS statistical software (SPSS 26.0; SPSS; Chicago, IL).

## Results

### In vitro study

At 50 ng/kg/min, the overall inhaled dose was similar between the high and low concentration delivery groups (11.68 ± 1.09 vs 11.20 ± 0.65 mcg, p = 0.693). At 30 ng/kg/min, the inhaled dose was greater in the high concentration group than the low concentration group regardless of weight (Table 1).

**Table 1:** In vitro comparisons of intermittent versus continuous delivery strategies with different case scenarios at iEPO doses of 50 and 30 ng/kg/min

iEPO dose, ng/kg/min	Cases of IBW	Nominal dose of iEPO in 20 min, mcg	Aerosol deposition of high concentration delivery			Aerosol deposition of low concentration delivery						p
			30 mcg/mL			15 mcg/mL			7.5 mcg/mL			
			Pump rate, mL/h	mcg	%	Pump rate, mL/h	mcg	%	Pump rate, mL/h	mcg	%	
50	50 kg	50	5.0	7.8 ± 0.8	15.5 ± 1.6	10.0	9.4 ± 0.4	18.8 ± 0.8	NA	NA	NA	0.050
	70 kg	70	7.0	12.2 ± 1.2	17.5 ± 1.7	14.0	10.6 ± 0.6	15.1 ± 0.9	NA	NA	NA	0.127
	90 kg	90	9.0	15.0 ± 0.9	16.7 ± 1.0	18.0	13.6 ± 0.8	15.1 ± 0.9	NA	NA	NA	0.127
	Overall	70	NA	11.7 ± 3.3	16.6 ± 1.5	NA	11.2 ± 2.0	16.4 ± 2.0	NA	NA	NA	0.691
30	50 kg	30	3.0	6.6 ± 0.6	21.9 ± 2.0	6.0	5.2 ± 0.5	17.5 ± 1.7	12.0	4.1 ± 0.3	13.6 ± 0.8	0.027
	70 kg	42	4.2	9.2 ± 1.1	22.0 ± 2.6	8.4	6.6 ± 0.8	15.8 ± 1.9	16.8	4.8 ± 0.5	11.4 ± 1.2	0.027
	90 kg	54	5.4	13.5 ± 1.0	25.0 ± 1.8	10.8	9.3 ± 1.8	17.3 ± 3.3	21.6	6.8 ± 0.2	12.6 ± 0.3	0.027
	Overall	42	NA	9.8 ± 3.1	23.0 ± 2.4	NA	7.1 ± 2.1	16.9 ± 2.2	NA	5.2 ± 1.3	12.5 ± 1.2	0.003

*iEPO* inhaled epoprostenol, *IBW* ideal body weight, *NA* not available

### Clinical study

#### Demographic information

**Fig. 2:**
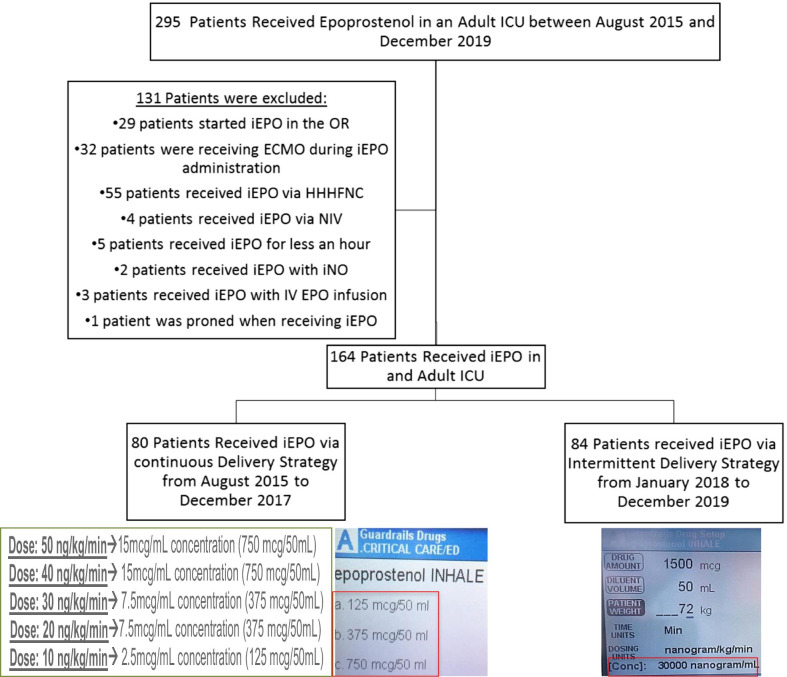
Flow chart of the two-period retrospective case–control study. In total 295 patients received iEPO in adult ICUs between August 2015 and December 2019. After excluding 131 patients, 164 patients were included for analysis. 80 patients received iEPO via low concentration delivery strategy from August 2015 to December 2017. With this strategy, three different concentrations of iEPO (750 mcg/50 mL, 375 mcg/50 mL, and 125 mcg/50 mL) and different pump rate were used, based on the prescribed dose (screenshot of the syringe pump was shown on the bottom left). 84 patients received iEPO via high concentration delivery strategy from January 2018 to December 2019. With this strategy, only one concentration (1500 mcg/50 mL) was used, and pump rate was adjusted to deliver different prescribed dose (screenshot of the syringe pump was shown on the bottom right). *iEPO* inhaled epoprostenol, *OR* operating room, *ECMO* extracorporeal membrane oxygenation, *HHHFNC* high-flow high humidity nasal cannula, *NIV* noninvasive ventilation, *IV EPO* intravenous epoprostenol, *ICU* intensive care unit, *iNO* inhaled nitric oxide

In total, 295 patients received iEPO at adult ICUs, 131 patients were excluded for receiving iEPO via high-flow nasal cannula (HFNC) (n = 55) or noninvasive ventilation (n = 4), iEPO was initiated during ECMO (n = 32) or cardiac surgical operation (n = 29), receiving simultaneous administration of intravenous epoprostenol (n = 3), iNO (n = 2), or prone positioning (n = 1), and iEPO was utilized for less than 30 min (n = 5). A total of 164 patients were enrolled, with 80 receiving low concentration iEPO from August 2015 to December 2017 and 84 receiving high concentration iEPO from January 2018 to December 2019 (Fig. 2). There was no significant difference in age, gender, ethnicity, or indications for iEPO between the two groups (Table 2).

**Table 2:** Comparisons of demographic information and outcomes in the groups of high versus low concentration delivery

	High concentration delivery	Low concentration delivery	p
No. of patients	84	80	
Age (years)	56.0 (44.3, 65.8)	55 (44.5, 68.8)	0.920
Male, %	35 (42%)	36 (45%)	0.753
Ethnicity, %			0.268
Caucasian	29 (35%)	36 (45%)	
African American	37 (44%)	31 (39%)	
Hispanic	15 (18%)	10 (13%)	
Asian	0	2 (3%)	
Other	3 (4%)	2 (1%)	
Indication to use iEPO, %			
Hypoxemia	58 (69%)	56 (70%)	1.0
Pulmonary hypertension	30 (36%)	25 (31%)	0.620
Right heart failure	20 (24%)	14 (18%)	0.342
No. of patients had positive response to iEPO, %			
No. of patients had mPAP reduction ≥ 10%	46% (10/22)	47% (7/15)	1.0
No. of patients had SpO_2_/F_I_O_2_ increment ≥ 10%	43% (25/58)	38% (21/56)	0.572
No. of patients had SpO_2_/F_I_O_2_ increment ≥ 20%	26% (15/58)	18% (10/56)	0.368
No. of patients had PaO_2_/F_I_O_2_ increment ≥ 10%	76% (34/45)	80% (28/35)	0.789
No. of patients had PaO_2_/F_I_O_2_ increment ≥ 20%	69% (31/45)	63% (22/35)	0.637
iEPO duration, hours	42.0 (17.9, 94.6)	48.1 (18.1, 85.8)	0.868
iEPO weaning for survived patients	53	45	
Followed all steps to wean, %	96% (51/53)	71% (32/45)	< 0.001
Extubated to iEPO via HFNC, %	17% (9/53)	2% (1/45)	0.038
Total number of syringes administered per patient	5 (3, 10)	12 (6, 22)	0.001
Duration of invasive ventilation, days	6 (3, 12)	9 (5, 18)	0.028
ICU length of stay, days	16 (8.3, 28)	15 (7.0, 30)	0.840
ECMO, %	10 (12%)	10 (13%)	1.0
Mortality, %	31 (37%)	35 (44%)	0.427

*iEPO* inhaled epoprostenol, *mPAP* mean pulmonary arterial pressure, *SpO*_*2*_ saturation of pulse oximetry, *F*_*I*_*O*_*2*_ fraction of inspired oxygen, *PaO*_*2*_ partial pressure of arterial oxygen, *ICU* intensive care unit, *ECMO* extracorporeal membrane oxygenation

#### Patient response to iEPO

114 patients received iEPO to treat refractory hypoxemia, 58 of them received high concentration delivery. After iEPO was initiated, oxygenation improved in both high and low concentration groups (p < 0.001). Of the 55 patients with pulmonary hypertension, 30 received high concentration iEPO. After iEPO was initiated, mPAP was significantly reduced in both high and low concentration delivery groups. Additionally, CO and CI were significantly improved in the high concentration group, in contrast to no significant pre- and post-change in the low concentration group. For the 20 patients with right heart failure who received high concentration iEPO, CO and CI were significantly improved following iEPO initiation. While for the 14 patients with right heart failure receiving low concentration iEPO, no significant differences of CO and CI were found pre- and post-iEPO delivery. Overall, no significant differences of incremental changes of oxygenation, mPAP, CO and CI were found between the two groups, regardless of the iEPO indications (Table 3). Regardless the iEPO response criteria, no significant differences of iEPO responders were found between two groups (Table 2).

**Table 3:** Comparisons of responses to iEPO between high and low concentration delivery strategies

iEPO indications	High concentration delivery			Low concentration delivery			p
	Pre	Post	p	Pre	Post	p	
Overall							
HR, beats/min	101.6 ± 20.7	100.7 ± 21.2	0.496	103.9 ± 19.6	102.7 ± 18.9	0.301	0.949
RR, breaths/min	24.0 ± 7.4	24.1 ± 6.4	0.923	25.5 ± 7.4	25.1 ± 7.3	0.373	0.964
mBP, mm Hg	77.3 (69.3, 85)	75.0 (68.0, 84.0)	0.158	74 (67.8, 84.5)	75 (70.0, 82.2)	0.669	0.773
Hypoxemia (n = 58 high concentration vs 56 low concentration)							
SpO_2_/F_I_O_2_	89.5 (84, 102.5)	99 (91.8, 134.6)	< 0.001	93.5 (88, 106.4)	98.5(93, 121.3)	< 0.001	0.436
PaO_2_/F_I_O_2_, mm Hg^a^	67(57, 101.4)	113.3 (78.9, 169.2)	< 0.001	73 (59, 88.8)	107.5 (77.8, 148.3)	< 0.001	0.948
PEEP, cm H_2_O	13.0 ± 4.2	13.3 ± 4.6	0.403	13.2 ± 5.0	12.9 ± 4.6	0.263	0.197
Pulmonary hypertension (n = 30 high concentration vs 25 low concentration)							
mPAP, mmHg^b^	39.1 ± 11.6	34.5 ± 10.3	0.012	36.7 ± 9.0	31.7 ± 7.3	0.019	0.551
CO, L/min^c^	4.2 ± 0.5	5.3 ± 1.1	0.003	6.3 ± 2.1	6.0 ± 1.8	0.635	0.337
CI, L/min/m^2 d^	2.22 ± 0.32	2.75 ± 0.70	0.006	3.23 ± 1.24	2.97 ± 0.92	0.593	0.231
SvO_2_, %^e^	65.6 ± 15.5	67.1 ± 15.4	0.131	65.2 ± 12.2	64.9 ± 8.3	1.0	0.429
Right heart failure (n = 20 high concentration vs 14 low concentration)							
mPAP, mmHg^f^	33.4 ± 14	29.8 ± 10.3	0.094	29.9 ± 7.1	27.6 ± 3.8	0.283	0.979
CO, L/min^g^	4.4 ± 0.6	5.5 ± 1.1	0.011	6.2 ± 3.1	6.2 ± 2.4	1.0	0.359
CI, L/min/m^2 h^	2.32 ± 0.37	2.89 ± 0.56	0.008	3.20 ± 2.06	2.95 ± 1.32	0.606	0.141
SvO_2_, %^i^	67.2 ± 10.8	68.2 ± 10.5	0.426	67.8 ± 14.7	65.8 ± 13.4	0.236	0.142

*iEPO* inhaled epoprostenol, *HR* heart rate, *RR* respiratory rate, *mBP* mean blood pressure, *mPAP* mean pulmonary arterial pressure, *SpO*_*2*_ saturation of pulse oximetry, *F*_*I*_*O*_*2*_ fraction of inspired oxygen, *PaO*_*2*_ partial pressure of arterial oxygen, *PEEP* positive end expiratory pressure, *CO* cardiac output, *CI* cardiac index, *SvO*_*2*_ saturation of mixed venous oxygen

^a^Data was available in 45 and 35 patients in intermittent and continuous delivery group, respectively

^b^Data was available in 22, 15 patients in two groups

^c^Data was available in 12, 9 patients in two groups

^d^Data was available in 13, 9 patients in two groups

^e^Data was available in 7, 10 patients in two groups

^f^Data was available in 14, 7 patients in two groups

^g^Data was available in 8, 4 patients in two groups

^h^Data was available in 9, 4 patients in two groups

^i^Data was available in 6, 4 patients in two groups

#### Adherence to iEPO weaning protocol and cost-effectiveness

Among the patients who survived, clinicians were more likely to be compliant with the iEPO weaning protocol (96% vs 71%, p < 0.001), and more patients were extubated to iEPO via HFNC (17% vs 2%, p = 0.038) in the high versus low concentration group. Additionally, the total number of syringes administered per patient was fewer in the high concentration group than the low concentration group (5 [3, 10] vs 12 [6, 22], p = 0.001) (Table 2).

#### Patient outcomes

No significant differences of iEPO duration, ICU mortality, escalation to ECMO, and ICU length of stay were found between the two groups. However, patients in the high concentration group had shorter duration of invasive ventilation (6 [3, 12] vs 9 [5, 18] days, p = 0.028) (Table 2).

## Discussion

This is the first study to compare high and low concentration iEPO delivery specific to adult mechanical ventilation. We found similar aerosol deposition and patient response of incremental changes in oxygenation and mPAP to high and low concentrations of iEPO during mechanical ventilation. However, we found that the high concentration group using one concentration and lower pump rates required fewer syringes and was associated with increased adherence to weaning per protocol. More importantly, it was associated with shorter duration of invasive ventilation.

The high concentration iEPO delivery only requires one syringe; therefore, medication preparation time is reduced. At our institution, with the high concentration delivery strategy, an iEPO syringe could be prepared ahead of time and stored in the refrigerator in the unit, making iEPO ready for use at any time. In contrast, as the low concentration iEPO delivery requires multiple concentrations and it is difficult to predict when the weaning or dose titration will be initiated, each syringe has to be made when the titration is ordered, the syringe is discarded when a new concentration is needed, resulting in medication waste. In our study, after switching to high concentration delivery of iEPO, we found lower total number of syringes per patient, which meant shorter medication preparation time.

When iEPO weaning is considered, the low concentration iEPO delivery requires multiple steps to wean. A new syringe of one of the three concentrations is prepared by pharmacy, which can ultimately delay the administration process. In contrast, weaning iEPO with high concentration delivery only requires reducing pump rate, which is easier to implement. The simple and quick application of high concentration delivery explains the higher adherence by clinicians to the weaning protocol in the high concentration group, which may also expedite weaning from invasive ventilation. The fast weaning of iEPO was especially crucial at the early phase of iEPO utilization in our institution, during which extubation would be implemented only when iEPO was weaned off. Our concerns were that if patients were directly extubated from iEPO and invasive ventilation to conventional oxygen therapy without iEPO, the loss of the effects of positive pressure and pulmonary vasodilation might cause rebound effects, due to the sudden increase in venous return to the heart, increased resistance of pulmonary vasculature, collapse of alveoli, and worsened oxygenation.

Since 2018, trans-nasal aerosol delivery to the lungs has attracted clinicians’ interest, and iEPO delivery via HFNC has been proven to be effective [15]. The feasibility of iEPO delivery via HFNC offered the option of extubating patients directly from invasive ventilation to HFNC with continuation of iEPO administration, which may allow patients capable of weaning from the ventilation without weaning off iEPO during invasive ventilation. We found more patients in the high concentration delivery group were extubated to receive iEPO via HFNC, which may also explain the shorter duration of invasive ventilation in our study.

We did not find significant differences of aerosol deposition between high and low concentration iEPO delivery in the in vitro study at 50 ng/kg/min. This likely explains the similar clinical response to iEPO in vivo. We did find a higher aerosol deposition with high concentration iEPO than low concentration iEPO at 30 ng/kg/min. High concentration iEPO had almost two-fold aerosol deposition of low concentration iEPO of 7.5 mcg/mL. The finding of higher delivery with high concentration iEPO agrees with our previous in vitro study performed in pediatric manikin during trans-nasal aerosol delivery [16]. However, the higher aerosol deposition with high concentration delivery was not reflected in clinical response, as we only recorded patients’ responses to iEPO when it was initiated at the dose of 50 ng/kg/min, which means we only compared patients’ responses to iEPO with concentrations of 15 and 30 mcg/mL, patients’ responses at low concentrations of 2.5 and 7.5 mcg/mL were not compared. Despite this, more efficient delivery at a lower ordered dose may be particularly important during weaning process, which might accelerate the iEPO weaning process. However, we did not find significant differences of ICU length of stay, this could be explained by the extended use of iEPO via HFNC post extubation that required patients to stay in ICU.

The use of a higher pump rate in the low concentration iEPO group produces high output of emitted aerosol, resulting in more aerosol losses or rain out and condensation in the ventilator circuit, increasing the resistance of expiratory filter [17]. Condensation in the circuit may cause aspiration if not being emptied in a timely manner, while the increased resistance in the expiratory filter may cause air-trapping in the patient’s lung or hemodynamic instability [18]. Therefore, with low concentration iEPO delivery, the ventilator circuit needs to be cleared and the expiratory filter needs to be replaced more frequently, requiring clinicians to break the mechanical ventilation circuit more often, which increase the risk of infection. In contrast, high concentration delivery can reduce emitted aerosol into the circuit, reducing the above risks.

The main limitation of this study was the retrospective nature and the lack of randomization. The retrospective design of the study was necessary to compare the outcomes of patients who were placed on iEPO using high and low concentration delivery strategies. We did not investigate other continuous inhaled medications, such as continuous albuterol, thus our findings could not simply apply for those medications, but it implies the demand for the future studies on those medications. For the patients with refractory hypoxemia, we only recorded the PEEP, PaO_2_/F_I_O_2_ ratio and SpO_2_/F_I_O_2_ ratio pre and post iEPO delivery, while some parameters such as ventilator mode and the use of neuromuscular blockade that might affect oxygenation were not recorded. However, considering the recording periods of 0.5–2 h before and 0.5–2 h after iEPO delivery, the possibility to have those parameters changed in the short window is low. Future studies reporting oxygenation responses to iEPO should include those parameters.

## Conclusion

Overall, our in-vitro and in-vivo study showed no significant difference in aerosol deposition or in patient's responses to iEPO with high vs low concentration delivery of iEPO for ICU patients receiving mechanical ventilation. However, a shorter duration of invasive ventilation was found in the high concentration delivery group, probably due to the faster iEPO weaning process, higher adherence to iEPO weaning, and more frequent use of extubation-to-iEPO via HFNC. We also found that fewer syringes were used in the high concentration delivery. Thus, all the clinical advantages support the use of high concentration iEPO over low concentration.

## Data Availability

Data are available upon reasonable request. Proposals should be directed to the corresponding author.

## Abbreviations

iEPO: Inhaled epoprostenol
VMN: Vibrating mesh nebulizer
PBW: Predicted body weight
Vt: Tidal volume
RR: Respiratory rate
PEEP: Positive end-expiratory pressure
F_I_O_2_: Fraction of inspired oxygen
Ti: Inspiratory time
ECMO: Extracorporeal membrane oxygenation
mPAP: Mean pulmonary arterial pressure
sPAP: Systolic pulmonary arterial pressure
CVP: Central venous pressure
CI: Cardiac index
mBP: Mean blood pressure
CO: Cardiac output
SvO_2_: Mixed venous oxygen saturation
PVR: Pulmonary vascular resistance
HR: Heart rate
SpO_2_: Saturation of pulse oximetry
ICU: Intensive care unit
SD: Standard deviation
IQR: Inter-quartile range
HFNC: High-flow nasal cannula
